# Ants Use Partner Specific Odors to Learn to Recognize a Mutualistic Partner

**DOI:** 10.1371/journal.pone.0086054

**Published:** 2014-01-29

**Authors:** Masaru K. Hojo, Ari Yamamoto, Toshiharu Akino, Kazuki Tsuji, Ryohei Yamaoka

**Affiliations:** 1 Faculty of Agriculture, University of the Ryukyus, Nishihara, Okinawa, Japan; 2 Graduate School of Science and Technology, Kyoto Institute of Technology, Kyoto, Japan; 3 Museum of Comparative Zoology, Harvard University, Cambridge, Massachusetts, United States of America; 4 The United Graduate School of Agricultural Sciences, Kagoshima University, Kagoshima, Japan; Sheffield University, United States of America

## Abstract

Regulation via interspecific communication is an important for the maintenance of many mutualisms. However, mechanisms underlying the evolution of partner communication are poorly understood for many mutualisms. Here we show, in an ant-lycaenid butterfly mutualism, that attendant ants selectively learn to recognize and interact cooperatively with a partner. Workers of the ant *Pristomyrmex punctatus* learn to associate cuticular hydrocarbons of mutualistic *Narathura japonica* caterpillars with food rewards and, as a result, are more likely to tend the caterpillars. However, the workers do not learn to associate the cuticular hydrocarbons of caterpillars of a non-ant-associated lycaenid, *Lycaena phlaeas*, with artificial food rewards. Chemical analysis revealed cuticular hydrocarbon profiles of the mutualistic caterpillars were complex compared with those of non-ant-associated caterpillars. Our results suggest that partner-recognition based on partner-specific chemical signals and cognitive abilities of workers are important mechanisms underlying the evolution and maintenance of mutualism with ants.

## Introduction

Cooperation between individuals of different species, called mutualism, is ubiquitous in nature but vulnerable to selfishness and cheating [1]–[3]. In such interactions, coordination of investments help stabilize the association, and thus an important factor underlying the maintenance of mutualism is the regulation of the relationship through interspecific communication [4]–[6].

Ants engage in mutualistic associations with various organisms, including plants and homopteran and lepidopteran insects, by exchanging defense in return for nutritious rewards [7]–[9]. However, nutritious rewards is costly for their partners and the quality of those rewards changes depending on their physiological state and environmental conditions [7], [9]–[14]. Thus natural selection may favor cognitive abilities in the ant that allow efficient recognition of cooperative partners. On the other hand, the ant’s partners compete for the protection provided by ant mutualists if they offer similar services (i.e. nutritious rewards composed by the mixture of sugars and amino acids) for ants [11], [15], [16]. Such competition for ants might then increase investment in advertisement of rewards which leads effective signal designs in ant’s partners [4], [17]. To explore these possibilities, we examined the recognition process and signals used in the mutualistic association between the lycaenid butterfly *Narathura japonica* and the ants *Pristomyrmex punctatus*.

More than half of lycaenid butterfly species are associated in some way with ants, ranging from casual co-existence to parasitism [8], [18]. Most associations are facultative rather than species-specific, obligate mutualisms. Typically, lycaenid caterpillars provide nutritious droplets in exchange for protection against enemies by workers from several different species of ants [19]. Lycaenid caterpillars have several specialized exocrine glands, which are used to signal to their associated ant partners [8], [20]. Caterpillars of all lycaenids are covered with single cell, epidermal glands called “pore cupolae” that are thought to secrete substances that appease associated ants [8], [18]. Some of caterpillars have a dorsal nectary organ (DNO) on the dorsum of the 7th abdominal segment, flanked by tentacle organs (TOs) on the 8th abdominal segment. The DNO secrete nutritious droplets for ants, and the TOs are assumed to secrete volatile substances that attract and alert ants [8], [21]. In addition to these ant-associated organs, many lycaenids also stridulate to communicate with their attendant ants [22], [23].

The Japanese Oak-Blue, *N. japonica* (Lepidoptera; Lycaenidae), has mutualistic associations with several ant species. In our field site, about 75% of caterpillars were tended by 8 different ant species, including the parthenogenetic ant, *P. punctatus* (Hymenoptera; Formicidae), the subject of this study (Table S1). To explore the partner recognition process, we examined the effect of *N. japonica* caterpillar experience on cooperative behavior of *P. punctatus* workers. We then compared the recognition process between mutualistic and non-mutualistic lycaenid species to determine whether the caterpillar’s signals are shaped to attract partner ants.

## Results and Discussion

### Ants Recognize Caterpillars as Partners Based on their Previous Experience of Reward Feeding

**Figure 1:**
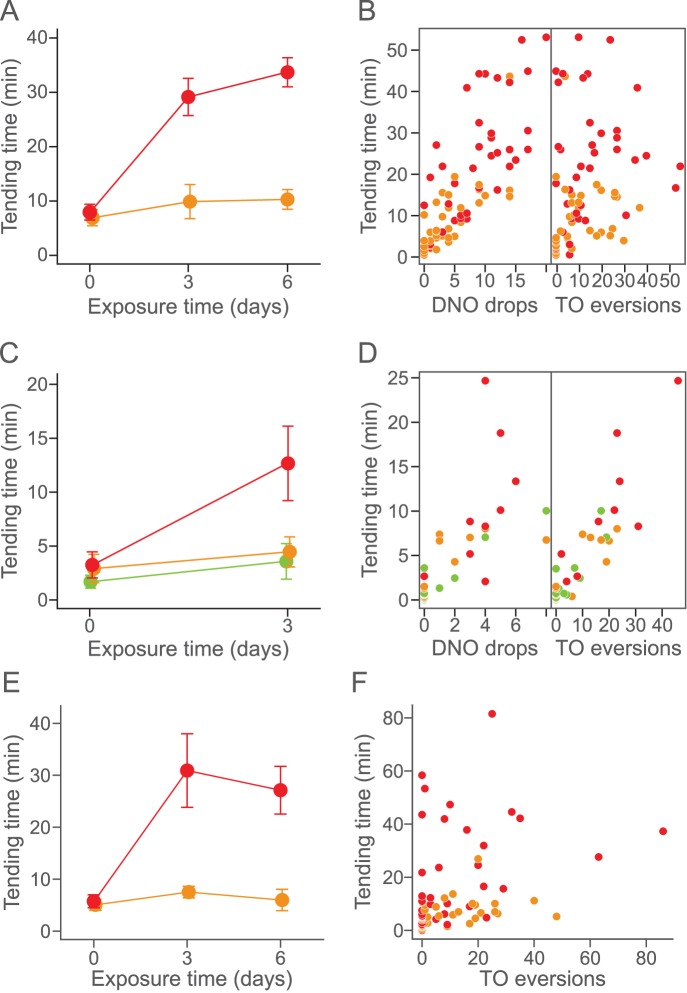
Prior exposure to secretions of *N. japonica* caterpillars induce prolonged tending behavior by *P. punctatus* workers. The standard error of the mean is shown. Red, orange and green circles indicate experienced, inexperienced and unrewarded treatments respectively. (A, B) Effect of prior exposure to caterpillars on ant tending behavior. (C, D) Effect of prior exposure to reward-less caterpillars on ant tending behavior. (E, F) Effect of prior exposure to the caterpillar on ant tending behavior toward reward-less caterpillars.

To explore the partner recognition process, we examined the effect of caterpillar experience on cooperative behavior in the laboratory. We maintained for 6 days, *P. punctatus* workers with a caterpillar of *N. japonica* (hereafter, these workers are called “experienced ants”). Another workers from the same colony were reared without exposure to a caterpillar, and used as controls (hereafter called “inexperienced ants”). On each of the first, third and sixth day, we conducted tending assays using 10 workers from each treatment and a new caterpillar (see material and methods section for details). We found that experienced ants were significantly more likely to tend caterpillars of *N. japonica* (Figure 1A; LR test, exposure time×treatment interaction, χ^2^ = 10.446, df = 1, p = 0.0053). Tending behavior was also correlated with the number of DNO drops (Figure 1B; χ^2^ = 29.079, df = 1, p<0.0001), but not with the number of TO eversions (Figure 1B; χ^2^ = 0.994, df = 1, *P = *0.3188). These results clearly indicated that, in addition to the number of DNO drops delivered, previous cooperative experiences with caterpillars are important in inducing tending behavior by ants.

A further experiment showed that reward feeding from the caterpillar was the key event inducing ant tending behavior (Figure 1C, D). In this experiment, we reared the ants with a caterpillar whose dorsal nectary organ was occluded with nail polish and thus could not secrete food droplets (hereafter, these workers are termed “unrewarded” ants associated with “reward-less” caterpillars, respectively). The results demonstrated that like inexperienced ants, unrewarded ants did not spend more time tending a novel, intact caterpillar (Figure 1C). *Post hoc* pair-wise comparisons among treatments revealed that the effect of exposure time×treatment interaction was significantly different between the experienced and inexperienced treatments (LR test with Bonferroni correction, p<0.05) and between experienced and unrewarded treatments (p<0.05). The exposure time×treatment interaction was statistically insignificant between inexperienced and unrewarded treatments (p>0.05). Unlike the results of the previous experiment (Figure 1B), however, the number of TO eversions was correlated with tending behavior in this experiment (Figure 1D; χ^2^ = 29.143, df = 1, p<0.0001), but the number of DNO drops was not (Figure 1D; χ^2^ = 2.949, df = 1, p = 0.0859). This discrepancy implies that the caterpillars of *N. japonica* can use both reward secretions and TO eversions to regulate ant attendance, but these two types of behavior are likely to be independent of each other as reported in another lycaenid species [21]. Taken together, our results demonstrate that the feeding on reward secretions from caterpillars is necessary to induce cooperative behavior by attendant ants.

### Cuticular Odors are used to Recognize a Mutualist Caterpillar

**Figure 2:**
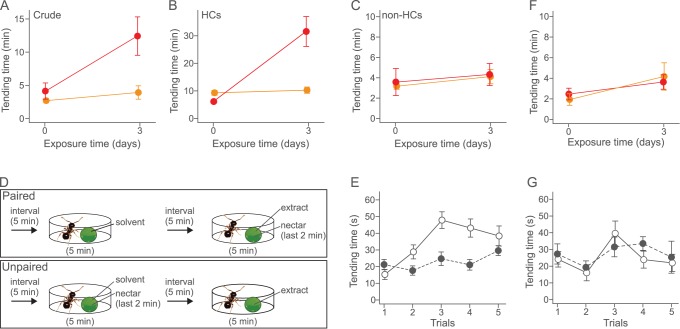
Cuticular hydrocarbons were used by *P. punctatus* workers to learn to recognize *N. japonica* caterpillars. The standard error of the mean is shown. Red and orange circles indicate the experienced and inexperienced treatments in (A-C and F). White and black circles indicate the paired and the unpaired treatments in (E) and (G). The effect of prior exposure to the caterpillar on ant tending behavior toward (A) the cuticular extract, (B) the hydrocarbon (HCs) fraction and (C) the non-HCs fraction. (D) The experimental protocol for each run of associative learning assays. (E) The time spent tending by workers trained with *N. japonica* cuticular odors paired with artificial secretions compared with that of workers trained with unpaired cuticular odors over 5 trials (n = 20). (F) The effect of prior exposure to caterpillars of *L. phlaeas* on ant tending behavior. (G) Tending time of workers trained with *L. phlaeas* cuticular odor paired with artificial secretions and with cuticular odor unpaired with artificial secretions over 5 trials (n = 10).

What is the nature of the signals used by caterpillars to induce tending behavior by the experienced ants? Possible signals are the reward secretion *per se*, which influences ant attendance of lycaenid larvae [21], [24], [25]. However, this hypothesis is unlikely given that unlike their experienced counterparts, inexperienced ants provided with reward secretions did not show a higher level of tending behavior (Figure 1A–D). To exclude the effect of the reward secretions on ant tending behavior, the rewardless-caterpillars were presented to both experienced and inexperienced workers. As expected, the experienced ants showed tending behavior toward reward-less caterpillars (Figure 1E,F, Movie S1, S2; exposure time×treatment interaction, χ^2^ = 11.868, df = 1, p = 0.0026, TO eversions, χ^2^ = 2.4594, df = 1, p = 0.1168). These results indicate that the experienced ants use not only the reward secretions, but also other larval signals to recognize partners. Ants use cuticular hydrocarbon composition to discern social information, including colony membership, task and fertility [26]–[28], and cuticular hydrocarbons have also been shown to mediate recognition between ants and their symbionts, including lycaenid caterpillars [29]–[33]. To test the idea that cuticular hydrocarbons are involved in the recognition between ants and lycaenid caterpillars in this system, we investigated the tending behavior of the ants toward glass dummies coated with cuticular chemicals of the caterpillars. The results showed that experienced ants were significantly more likely to tend glass beads coated with crude cuticular chemicals (Figure 2A; exposure time×treatment interaction, χ^2^ = 4.897, df = 1, p = 0.0269) and hydrocarbon fractions (Figure 2B; χ^2^ = 16.708, df = 1, p<0.0001) extracted from caterpillars than to control glass dummies (Figure S1) or dummies coated with the non-hydrocarbon fraction (Figure 2C; χ^2^ = 0.011, df = 1, p = 0.9133). These results indicated that the experienced *P. punctatus* workers use cuticular hydrocarbons to recognize *N. japonica* caterpillars.

Recent evidence has demonstrated that cuticular hydrocarbons are detected by chemosensilla in the antennae that have trajectories to the primary olfactory center of the brain [34]–[36]. Lycaenid secretions containing carbohydrates and amino acids are perceived by gustatory receptor cells on the taste sensilla [37], [38]. These findings suggest that ants learn to associate lycaenid secretions with cuticular hydrocarbons of *N. japonica* caterpillars, and the combined signal elicits tending behavior. To test this, we conducted associative learning assays using artificial secretions and cuticular extracts of *N. japonica* caterpillars. Chemical analyses revealed that the secretions of *N. japonica* consisted of a mixture of 3 sugars and 19 amino acids. Based on this result, we made an artificial secretion that was used for learning assays (Table S2). In the learning assay, we alternately presented a control dummy and a dummy coated with cuticular chemicals of *N. japonica* to naïve workers of *P. punctatus* that had never contacted a caterpillar of *N. japonica* before (Figure 2D). Over 5 successive conditioning trials, the tending time of workers toward dummies increased in the paired treatment, but did not change in the unpaired treatment (Figure 2E; trial×treatment interaction, χ^2^ = 8.286, df = 1, p = 0.0039). These results indicate that the ant tending behavior toward the caterpillars is based on simple associative learning of secreted rewards and cuticular hydrocarbon profiles of the caterpillars.

### Ants do not use Cuticular Odors of a Non-ant-associate as Recognition Cues

Insect cuticular hydrocarbons are used by most insects to prevent water loss [39]. Thus non-ant-associated lycaenid butterflies also secrete hydrocarbons on their cuticles. If *N. japonica* hydrocarbons have been selected to enhance detectability by attendant ants according to the sensory bias of those ants, *N. japonica* cuticular hydrocarbons will be more efficiently learned by the *P. puctatus* ant partner than the cuticular hydrocarbons of a non-ant-associate lycaenid species. To test this hypothesis, we investigated the effect of previous experience with a non-ant-associated caterpillar on the ant tending behavior using another lycaenid butterfly species, *Lycaena phlaeas.* The caterpillars of *L. phlaeas* lack both the dorsal nectary organ and tentacle organs, and are not tended by ants in the field and laboratory [40]. When we reared the workers with the *L. phlaeas* caterpillars, experienced ants did not spend more time tending the *L. phlaeas* caterpillar than inexperienced ants (Figure 2F; exposure time×treatment interaction, χ^2^ = 0.549, df = 1, p = 0.4587). In addition, the associative learning experiments using artificial secretions based on *N. japonica* secretions and *L. phlaeas* cuticular chemicals revealed that the tending behavior of the ants did not differ between paired and unpaired treatments (Figure 2G; trial×treatment interaction, χ^2^ = 0.004, df = 1, p = 0.9934). The amount of time that workers spent tending dummy models in paired treatments was also significantly different between models treated with *N. japonica* versus *L. phlaeas* cuticular chemicals (χ^2^ = 6.895, df = 1, p = 0.0086). These results indicated that the cuticular hydrocarbons of *N. japonica* function as signals for *P. punctatus* workers, and thus the workers could more efficiently learn to associate rewards when paired with the cuticular hydrocarbons of *N. japonica* than with *L. phlaeas*.

**Figure 3:**
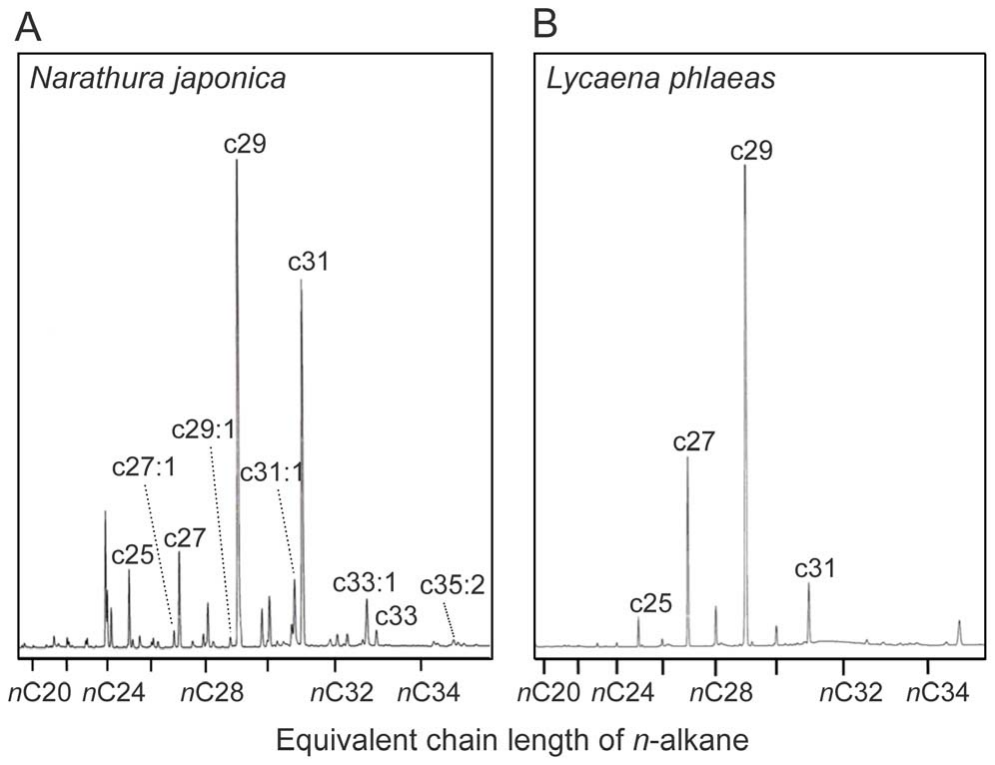
Representative chromatograms of hexane extracts of the mutualistic *N. japonica* and non-ant-associated *L. phlaeas* caterpillars. (A) *Narathura japonica* hydrocarbons consisted of a mixture of *n*-alkanes, *n*-alkenes and *n*-alkadienes, whereas (B) *L. phlaeas* hydrocarbons mainly consisted of *n*-alkanes (see also Table S3).

Analyses of cuticular hydrocarbons of both lycaenid species revealed that the total amount of hydrocarbons of *N. japonica* (4.00±1.49 µg: n = 10: mean ± s.e.m.) and *L. phlaeas* (5.53±1.16 µg: n = 11: mean ± s.e.m.) were not significantly different (t-test; t = 0.809, df = 17.459, p = 0.429), but the qualitative traits of the hydrocarbons differed substantially between the two lycaenid species. Caterpillars of *L. phlaeas* have a simple set of hydrocarbons, mainly composed of *n*-alkanes (Figure 3B) but *N. japonica* caterpillars have a complex mixture of *n*-alkanes, *n*-alkenes and *n*-alkadienes (Figure 3A and Table S3). These results suggest that the behavioral differences exhibited by the ants were not caused by a difference in the amount of hydrocarbons, but rather on their qualitative traits. Because cuticular hydrocarbons of *P. punctatus* contain various unsaturated hydrocarbons (Table S4), it is likely that *P. punctatus* use these compounds to communicate with nest-mate, and thus the unsaturated hydrocarbon class would be easily detected by the workers. Several studies have shown that the ant workers appear to use specific hydrocarbon classes in nest-mate recognition cues [41]–[44]. It is also known that the ability of social insects to learn hydrocarbon profiles is affected by the structure of the hydrocarbons [45]–[47]. Further learning assays using synthetic hydrocarbons and comparative studies using different lycaenid species are needed to understand the importance of hydrocarbon classes on recognition and maintenance of ant associations.

## Conclusions

Previous studies of ant-protection mutualisms have reported that the ant partners can change the rates, concentration and composition of their reward secretions depending upon the need to regulate ant attendance [10], [21], [24], [25], [48], [49]. We showed that learning to associate the cuticular hydrocarbon profile with food rewards plays an important role in mediating cooperative behaviors between ants and their symbionts. Because the quality of lycaenid secretions varies depending on various biotic and abiotic factors, learning to associate the hydrocarbon profile with reward secretions should serve as a good indicator of the quality of the partner species for the attendant ants. Several ant species can learn to recognize cuticular hydrocarbons [45], [46], and a recent study reported that cuticular hydrocarbons are used as recognition cues in ant-aphid mutualisms [50]. We believe that the regulation of the relationship with ants based on associative learning of cuticular hydrocarbons is not restricted to associations with lycaenids, but may be common among various ant-protection mutualisms.

For non-ant-associated lycaenids, avoiding attack from ants is nevertheless highly adaptive, and the caterpillar’s simple hydrocarbon profiles might reflect this function, since *n*-alkanes induce less aggressive behavior by some ant species [41]–[43]. For mutualistic partners, advertising by investing in more complex hydrocarbon signals is useful to attract attendant ants, especially when there is competition for ant partners. We suggest that variation in hydrocarbon profiles of lycaenid larvae may reflect degree of ant association. At the same time, selection may have also favored ant sensory systems that can recognize and efficiently learn the odors of the most profitable lycaenid partners. Many species of lycaenid butterflies have evolved complex adaptations enabling them to live in association with ants [8], [18]. Further analyses of lycaenid signalling and perception by ants will provide valuable insight into the evolution of interspecific communication in mutualisms.

## Materials and Methods

### Study Organisms

*Narathura japonica* (Theclinae) is native to oak woods in Japan, Taiwan and Korea. Caterpillars feed on species of *Quercus* (Fagaceae) and are usually associated with ants. We collected eggs and early instar caterpillars feeding on *Q. glauca* in Kyoto city from 2007 to 2009, and in Okinawa in 2011, and reared them on young leaves of *Q. glauca*. Early instar caterpillars of *Lycaena phlaeas daimio* (Lycaeninae) were collected at Kyoto city in 2009, and reared on cuttings of their host plant, *Rumex japonicus*. Colonies of the ant, *Pristomyrmex punctatus* have no queen, but workers reproduce through parthenogenesis. We collected three colonies of *P. punctatus* in Kyoto in 2007 and three colonies in Okinawa in 2011. All colonies were collected in areas that did not contain host plants of *N. japonica*. Therefore, to the best of our knowledge, the ants had never previously encountered caterpillars of *N. japonica*. No specific permit was required to collect these ant and butterfly species, which is not endangered or protected. The ants were reared in a plastic nest box (70×50×40 cm) with nest materials (rotting leaves and wood). Mealworms, maple syrup solution and Bhatkar-Whitcomb diet [51] were provided as food in the foraging area twice each week.

### Field Surveys of Ant Attendance

We surveyed 4 sites near Kyoto city on ten occasions from May to October in 2009. We investigated every young leaf on *Q. glauca* hedges at every site and recorded the number of *N. japonica* caterpillars and tending ant species. We did not measure the caterpillar’s instar. Each observation was made in the afternoon (1PM to 6PM).

### The Effect of Exposure to Caterpillars on ant Tending Behavior

150 workers from the foraging area of each colony were collected and kept in a plastic arena (17×12×5 cm) containing food in the form of cotton soaked in10% sugar solution, water and young leaves of *Q. glauca*. For the “experienced” treatment, a 5th (final) instar caterpillar was introduced to the arena with the foraging ants. Another 150 workers from the same colony were reared under similar conditions but without a caterpillar, and these were used as the “inexperienced” treatment. The caterpillars had never been exposed to other ants before being used in the experiment. Tending assays (see below) were conducted soon after the introduction on Day 0 (when workers from both treatments had not yet contacted the caterpillar.), Day 3 and Day 6 (conducted for *N. japonica* only). (for *N. japonica*, n = 13, using 6 ant colonies in each treatment; for *L. phlaeas*, n* = *6 using 3 ant colonies in each treatment). Rearing cases were checked and cleaned every day, and if a caterpillar became a pre-pupa, we replaced it with another 5th instar caterpillar. This exchange did not interrupt the feeding condition treatment of ants because preliminary observation confirmed that *N. japonica* pre-pupae also secrete food rewards and are tended by ants (Hojo MK, personal observation).

### The Effect of Exposure to Caterpillars Plus Reward Secretions on Ant Tending Behavior

Caterpillars designated as “reward-less” were experimentally manipulated by applying a small amount of clear nail polish on and around the dorsal nectary organ of 5th instar caterpillars. To make sure that the dorsal nectary organ was successfully occluded, the caterpillar’s ability to produce rewards was subsequently checked under the microscope. Preliminary observation confirmed that the nail polish was sufficient to prevent secretion. We also confirmed that observed both ant and caterpillar behaviors were not disrupted by the nail polish application, by putting nail polish on other areas of the caterpillars. The experienced and inexperienced treatments were produced as described above, and an additional 150 workers from the same colony were reared with the reward-less caterpillar ( = “unrewarded” treatment). Tending assays using the intact caterpillars were conducted on Day 0 and Day 3 (n* = *6 using 3 colonies in each treatment).

### The Effect of Reward Secretion on Ant Tending Behavior

The experienced and inexperienced treatments are the same as those described above. Tending assays using reward-less caterpillars were conducted on Day 0, Day 3 and Day 6 days (n* = *12 using 6 colonies in each treatment).

### Tending Assay

Ten workers were randomly chosen from a nest box and moved to a plastic petri dish (4.5 cm i.d.×1 cm height) where they were allowed to become familiar with their environment for 15 minutes. A fresh caterpillar (intact or reward-less, depending on the experiment) was introduced to the petri dish, and behavioral interactions were recorded for 15 min using a video camera (IXY DV M5, Canon). We measured three behavioral responses: (1) the total time tended by ants, measured in ant min (i.e. two ants tending for 30 sec each = 1 ant min), (2) the number of secretion droplets produced by the caterpillar for the ants, and (3) the number of the TO eversions produced by the caterpillars. After each assay, the workers were not returned to the original colony or nest boxes to avoid social learning. We used different individual caterpillars for each tending assay.

### The Effect of Prior Exposure to a Caterpillar on Ant Response Toward Cuticular Chemical Profiles

To make the cuticular chemical extract, a caterpillar was immersed in approximately 2 ml of *n*-hexane for 2 min. For hydrocarbon and non-hydrocarbon fractions, the crude extract was chromatographed on ca. 0.2 g of silica gel (230–400 mesh ASTM, 0.040–0.063 mm, Merck Ltd., Germany). Hydrocarbons were eluted with 2 ml of *n*-hexane and non-hydrocarbon compounds were eluted with 2 ml of methylene chloride. The extracts and fractions were concentrated and re-dissolved in 40 µl of *n*-hexane. 20 µl (0.5 caterpillar equivalent) was applied to a green flat glass bead (8 mm diameter×2 mm height). The extracts from different individual caterpillars were used for each assay. A glass bead treated with 20 µl *n*-hexane was used as a control. All beads were used after the evaporation of solvent (approximately 30 min after application). The experienced and the inexperienced ants were produced as described above using 50 workers in each treatment (n = 6 for each crude and control experiments using 3 colonies; n = 9 for the hydrocarbon and non-hydrocarbon fraction experiments using 3 colonies). After the 0 and 3 days, the glass bead coated with cuticular extract was gently placed on the center of the petri dish (4.5 cm i.d.×1 cm height) containing 10 experienced or 10 inexperienced workers without disturbance and the ant behavior towards dummies were videotaped for 15 min. We measured tending time (ant min) toward the glass dummy and compared this for experienced and inexperienced ants.

### Secretion Collection and Analyses

A 5th instar caterpillar of *N. japonica* and five workers of *P. punctatus* were introduced to a glass petri dish (4 cm i.d.×2 cm height). When the workers antennated the dorsal nectary organ, the caterpillar secreted a droplet. These droplets were collected using 0.5 µl microcapillaries (MICROCAPS; Drummond, Broomall, PA, USA). Immediately after collection, all samples in the microcapillaries were stored at –30°C until HPLC analysis.

For the sugar analysis, samples (0.3–1.2 µl) were added to 15 µl of Milli-Q-Water and 10 and 5 µl aliquots were used for sugar and amino acid analysis, respectively. For sugar analysis, samples were analysed by high performance liquid chromatography using a 5NH_2_-MS packed column (4.6×150 mm; Cosmosil, Nacalai Tesque, Kyoto, Japan) at room temperature. The mobile phase was 80% acetonitrile (Wako Chemicals, Tokyo, Japan) and the flow rate was 1 ml/min. The samples were injected directly into the column. Peak sizes for the sugars present in the samples were calculated directly using a refractive index detector (RID6A; Shimadzu, Kyoto, Japan) and these were used to estimate the concentrations of sugars in the caterpillar secretions. The sugars in the secretions were identified by comparison with the retention times of standard sugar solutions (d-xylose, d-fructose, d-glucose, d-galactose, sucrose, turanose, maltose, trehalose, lactose, melibiose, melezitose, and raffinose; all from Nacalai Tesque, Kyoto, Japan).

For amino acid analysis, each sample was adjusted with 0.02 N HCl (Nacalai Tesque, Kyoto, Japan) to a final volume of 100 µl, and analyzed using an automated amino acid analyzer L-8800 (Hitachi, Tokyo, Japan).

### Learning Assay

The appetitive unconditioned stimulus (US) was the artificial secretion, which contained sugars and amino acids in roughly the same concentrations detected in the secretions of *N. japonica* (Table S2). The conditioned stimulus was the hexane extract of the cuticular chemicals from a caterpillar of *N. japonica* caterpillar or *L. phlaeas*. Each trial lasted 20 min and was composed of two experimental sections, and two blanks (Figure 2C). In the paired conditioning, a worker was collected from the foraging area and moved to a glass petri dish (4 cm i.d.×2 cm height) and left for 5 min to equilibrate (the first blank). In the first experimental section, the green flat glass bead that had been coated with 20 µl *n*-hexane (i.e., a control bead) was placed in the center of the dish. The section lasted 5 min, whereupon the bead was removed and the second experimental section was started for another 5 min (the second blank). In the second experimental section, the glass bead coated with 0.5 of a caterpillar’s equivalent of cuticular chemical (i.e., an extract bead) was put in the center of the dish. This was carried out with the extracts of two different caterpillar species, *N. japonica* and *L. phlaeas*. The tending time was measured for the first 3 min, after which a small amount (<0.1 µl) of artificial secretion was gently placed on the center of the glass bead using a 0.5 µl micro capillary (MICROCAPS; Drummond, Broomall, PA, USA). The ants could feed freely on the artificial secretion for the subsequent 2 min while contacting the glass bead. The bead was removed after each section. The whole procedure was replicated 5 times in succession, and thus the total experimental time was 100 min. In the unpaired conditioning runs, the both extract and control glass beads were presented in the same way as in the paired conditioning runs, but the artificial secretion was placed on the center of the control beads (i.e. the first experimental section). Thus the ants in both treatments, paired and unpaired, were subjected to 5 control bead sections (5×5 min), 5 extract bead sections (5×3 min), 5 secretion feeding sections (5×2 min), and 10 blanks (10×5 min), but only the workers of the paired treatment were simultaneously presented with the cuticular extract and the artificial secretions.

### Cuticular Hydrocarbon Analyses

GC analyses were carried out using a GC-2014 (Shimadzu, JAPAN) with a flame ionization detector (FID). The GC was fitted with a DB-1 column (30 m×0.25 mm×0.15 µm; J &W Scientific, USA), programmed from 60°C for 1 min, 20°C/min to 260°C, 5°C/min to 300°C and held for 11 min. Helium was used as a carrier gas and the column head pressure was 100 kPa. The samples were injected with an internal standard (*n*-octadecane). Injection and detector were at 300°C. Data were collected and calculated using the CR-8A Chromatopac Data Processor (Shimadzu, JAPAN). Representative samples were analysed by GC-MS using a GC-17A (Shimadzu, Japan) interfaced to a QP-5050 quadrupole mass spectrometer (Shimadzu, Japan) in EI mode with 70 eV. The MS interface temperature was 300°C and GC conditions were the same for GC-FID analysis.

### Data Analysis

The tending assay data were analyzed using a linear mixed model (LMM). A model was constructed using tending time as a response variable with an identity link function, group ID nested within ant colony as a random effect (random intercept), and exposure time (days), treatment (inexperienced, experienced and unrewarded), number of DNO drops delivered (not used in “The effect of reward secretion on ant tending behavior”), number of TO eversions and exposure time×treatment interactions as fixed effects. For the learning assay, a model was constructed using tending time as a response variable with an identity link function, individual identity nested within colony as a random intercept, and treatment (paired, unpaired) and trial (trial 1 to 5) as fixed effects. For each analysis, the influence of different factors were tested using the likelihood ratio (LR) test. In multiple comparisons, the data containing two compared factors were extracted from the complete data set and fitted by LMM in a similar way, and then the influence of factors were tested using the LR test with Bonferroni correction. All statistical analyses were conducted in R version 2.14.0 [52].

## Supporting Information

Figure S1 **The effect of prior exposure to caterpillars of** ***Narathura japonica*** **on ant tending behaviour toward solvent-treated glass dummies.** The effect of the time×treatment interaction was not significant (LR test, *n* = 6, χ^2^ = 0.127, df = 1, *p* = 0.7214). The standard error of the mean is shown. Red and orange circles indicate experienced and inexperienced treatments respectively.
(PDF)

Table S1 **Ant species observed tending caterpillars of** ***N. japonica*** **in Kyoto city from May to October 2009.**
(PDF)

Table S2 **Sugar (** ***n*** **= 14) and amino acid (** ***n*** **= 13) composition of the larval secretions of** ***N. japonica*** **(mean and standard error) and the artificial secretions.**
(PDF)

Table S3 **Relative amount (mean and standard error) of cuticular hydrocarbons of mutualistic** ***N. japonica*** **(** ***n*** **= 10) and non-ant-associated** ***L. phlaeas*** **(** ***n*** **= 11).**
(PDF)

Table S4 **List of cuticular hydrocarbons of** ***Pristomyrmex punctatus.***
(PDF)

Movie S1 **Tending behavior of the inexperienced ants toward the “reward-less” caterpillar.**
(MOV)

Movie S2 **Tending behavior of the experienced ants toward the “reward-less” caterpillar.**
(MOV)
