# Supplementary figures and images for: Ants Use Partner Specific Odors to Learn to Recognize a Mutualistic Partner
Source: PLoS One. 2014 Jan 29;9(1):e86054. doi: 10.1371/journal.pone.0086054 (PMC3906017; doi:10.1371/journal.pone.0086054)

**Supporting information:**

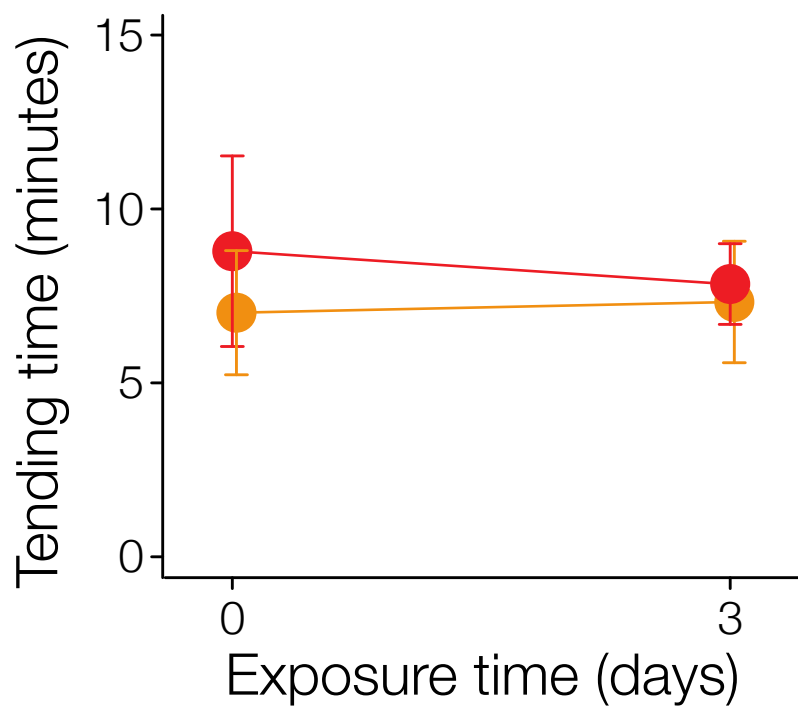

Supplement: Figure S1 — The effect of prior exposure to caterpillars of Narathura japonica on ant tending behaviour toward solvent-treated glass dummies. The effect of the time×treatment interaction was not significant (LR test, n = 6, χ2 = 0.127, df = 1, p = 0.7214). The standard error of the mean is shown. Red and orange circles indicate experienced and inexperienced treatments respectively. (PDF) [file pone.0086054.s001.pdf]
