# Supplementary material for: Ants Use Partner Specific Odors to Learn to Recognize a Mutualistic Partner
Source: PLoS One. 2014 Jan 29;9(1):e86054. doi: 10.1371/journal.pone.0086054 (PMC3906017; doi:10.1371/journal.pone.0086054)
Supplement: Table S1 — Ant species observed tending caterpillars of N. japonica in Kyoto city from May to October 2009. (PDF) [file pone.0086054.s002.pdf]

Supporting information:

Table S1 Ant species observed tending caterpillars of *N. japonica* in Kyoto city from May to October 2009

Ant species	Number of caterpillars	Proportion (%)
Myrmicinae		
Crematogaster osakensis	4	1.57
Crematogaster sp.	47	18.50
Temnothorax congruus	3	1.18
Tetramorium tsushimae	1	0.39
Pristomyrmex punctatus	40	15.74
Formicinae		
Camponotus vitiosus	61	24.01
Camponotus japonicus	2	0.78
Lasius japonicus	33	12.99
Not tended	63	24.80
Total	254	100
