# Supplementary material for: Ants Use Partner Specific Odors to Learn to Recognize a Mutualistic Partner
Source: PLoS One. 2014 Jan 29;9(1):e86054. doi: 10.1371/journal.pone.0086054 (PMC3906017; doi:10.1371/journal.pone.0086054)
Supplement: Table S2 — Sugar ( n = 14) and amino acid ( n = 13) composition of the larval secretions of N. japonica (mean and standard error) and the artificial secretions. (PDF) [file pone.0086054.s003.pdf]

Supporting information:

Table S2. Sugar (n = 14) and amino acid (n = 13) composition of the larval secretions of *Narathura japonica* (mean and standard error) and the artificial secretions.

	N. japonica secretion		Artificial secretion
	mmol/L	g/L	g/L
Sugars			
D-fructose	53.24 ± 11.32	9.59 ± 2.04	10
D-glucose	55.68 ± 13.22	10.03 ± 2.38	10
sucrose	17.24 ± 8.10	5.90 ± 2.77	6
total	116.31 ± 24.49	28.43 ± 7.15	26
Amino acids			
L-glutamine	101.11 ± 20.29	14.778 ± 2.965	15
L-serine	16.69 ± 2.27	1.753 ± 0.238	2
L-arginine	14.99 ± 1.87	2.610 ± 0.324	2
L-phenylalanine	10.32 ± 0.88	1.704 ± 0.144	2
L-leucine	10.09 ± 0.99	1.323 ± 0.129	1.5
L-histidine	9.60 ± 1.22	1.488 ± 0.189	1.5
L-alanine	7.24 ± 0.93	0.645 ± 0.083	1
L-valine	3.33 ± 0.40	0.390 ± 0.047	0.4
L-glutamic acid	2.63 ± 0.58	0.386 ± 0.085	0.4
L-asparagine	2.30 ± 0.41	0.304 ± 0.054	0.3
L-methionine	1.89 ± 0.19	0.282 ± 0.028	0.3
glycine	1.70 ± 0.20	0.127 ± 0.014	0.1
L-threonine	1.67 ± 0.26	0.198 ± 0.030	0.2
L-lysine	1.64 ± 0.25	0.239 ± 0.036	-
L-iso-leucine	1.52 ± 0.21	0.199 ± 0.027	0.2
L-aspartic acid	1.47 ± 1.09	0.195 ± 0.145	-
L-proline	1.33 ± 0.44	0.153 ± 0.050	-
L-tyrosine	1.21 ± 0.22	0.219 ± 0.040	0.2
L-cystein	0.13 ± 0.01	0.016 ± 0.002	-
Total	188.95 ± 23.83	26.875 ± 3.433	27.1
