# Supplementary material for: Ants Use Partner Specific Odors to Learn to Recognize a Mutualistic Partner
Source: PLoS One. 2014 Jan 29;9(1):e86054. doi: 10.1371/journal.pone.0086054 (PMC3906017; doi:10.1371/journal.pone.0086054)
Supplement: Table S3 — Relative amount (mean and standard error) of cuticular hydrocarbons of mutualistic N. japonica ( n = 10) and non-ant-associated L. phlaeas ( n = 11). (PDF) [file pone.0086054.s004.pdf]

Supporting information:

Table S3 Relative amount (mean and standard error) of cuticular hydrocarbons of mutualistic *N. japonica* (*n* = 10) and non-ant-associated *L. phlaeas* (*n* = 11)

Substance	Retention index	Substance class	*N. japonica*	*L. phlaeas*
*n*C24	24.00	*n*-alkane	1.97 ± 0.27	-
*n*C25	25.00	*n*-alkane	2.91 ± 0.36	3.22 ± 0.36
unknown	25.13	unknown	3.14 ± 0.58	-
unknown	25.14	unknown	-	0.26 ± 0.01
*n*C26:1	25.95	*n*-alkene	1.87 ± 0.37	-
*n*C26	26.00	*n*-alkane	-	1.28 ± 0.21
unknown	26.13	unknown	0.81 ± 0.11	-
*n*C27:1	26.78	*n*-alkene	3.98 ± 0.49	-
*n*C27	27.00	*n*-alkane	5.89 ± 0.28	17.02 ± 1.09
*n*C28:1	27.84	*n*-alkene	3.65 ± 0.23	-
*n*C28	28.00	*n*-alkane	1.12 ± 0.20	4.10 ± 0.17
*n*C29:1	28.69	*n*-alkene	0.73 ± 0.09	-
*n*C29	29.00	*n*-alkane	35.47 ± 1.97	61.72 ± 1.34
unknown	29.20	unknown	-	0.98 ± 0.16
*n*C30:1	29.75	*n*-alkene	0.98 ± 0.10	-
*n*C30	30.00	*n*-alkane	3.76 ± 0.20	2.45 ± 0.22
*n*C31:1	30.76	*n*-alkene	4.35 ± 0.55	-
*n*C31	31.00	*n*-alkane	16.24 ± 0.79	7.07 ± 0.53
*n*C32:1	31.77	*n*-alkene	1.36 ± 0.15	-
*n*C32	32.00	*n*-alkane	1.33 ± 0.21	1.86 ± 0.25
*n*C33:2	32.41	*n*-alkadiene	0.54 ± 0.11	-
*n*C33:2	32.53	*n*-alkadiene	0.79 ± 0.18	-
*n*C33:1	32.68	*n*-alkene	2.46 ± 0.57	-
*n*C33	33.00	*n*-alkane	2.87 ± 0.24	-
unknown	34.43	unknown	0.77 ± 0.14	-
*n*C35:2	34.52	*n*-alkadiene	3.03 ± 0.32	-
