# Supplementary material for: Ants Use Partner Specific Odors to Learn to Recognize a Mutualistic Partner
Source: PLoS One. 2014 Jan 29;9(1):e86054. doi: 10.1371/journal.pone.0086054 (PMC3906017; doi:10.1371/journal.pone.0086054)
Supplement: Table S4 — List of cuticular hydrocarbons of Pristomyrmex punctatus. (PDF) [file pone.0086054.s005.pdf]

Supporting information:

Table S4 List of cuticular hydrocarbons of *Pristomyrmex punctatus*

No.	Compounds	Substance class
1	nC25:1	alkene
2	nC25	alkane
3	11MeC25, 13MeC25	branched alkane
4	7MeC25	branched alkane
5	5MeC25	branched alkane
6	3MeC25	branched alkane
7	5,10-, 5-12-, 5,13-, and/or 5,15-diMeC25	branched alkane
8	nC26	*n*-alkane
9	5MeC26	branched alkane
10	nC27:2	alkene
11	nC27:1	alkene
12	nC27	*n*-alkane
13	11Me, 13MeC27	branched alkane
14	7MeC27	branched alkane
15	5MeC27	branched alkane
16	3MeC27	branched alkane
17	5,12- and/or 5,15-diMeC27	branched alkane
18	nC28	*n*-alkane
19	nC29	*n*-alkane
20	C35:1	alkene
21	C36:1	alkene
22	C38:1	alkene
23	C38:2	alkene
24	C37:1	alkene
25	C37:4	alkene
26	C37:3	alkene
27	C37:2	alkene
28	C37:2	alkene
29	C39:3	alkene
30	C39:2	alkene
31	C39:1	alkene
32	C40:2	alkene
33	C41:4	alkene
34	C41:3	alkene
35	C41:2	alkene
36	C42:2	alkene
37	C43:4	alkene
38	C43:3	alkene
39	C43:2	alkene
40	C44:2	alkene
41	C45:4	alkene
42	C45:3	alkene
43	C45:2	alkene
